# Evolution of the Global Landscape of Nanoparticles and Nanotubes in Advancing Oncological Care: A Bibliometric Analysis

**DOI:** 10.7759/cureus.99102

**Published:** 2025-12-13

**Authors:** Tori M Baer, Rishank Chillakuru, Latha Ganti

**Affiliations:** 1 Biology, Oviedo High School, Oviedo, USA; 2 Biomedical Engineering, Case Western Reserve University, Cleveland, USA; 3 Medical Science, The Warren Alpert Medical School of Brown University, Providence, USA

**Keywords:** bibliometric analysis, cancer, nanocarrier drug delivery, nanomedicine, nano-oncology, nanoparticles, nanotechnology, nanotubes, oncology, precision technology

## Abstract

This study aims to analyze the literature published regarding nano-oncology and, more specifically, global trends concerning nanoparticles and nanotubes from 2003 to 2025. This bibliometric analysis gathered data from the Web of Science Core Collection, incorporating keywords such as oncology, nanotechnology, nanomedicine, nanoparticles, and nanotubes. With the retrieved data visualized in the VOSViewer program and Web of Science Core Collection, the primary focus was on keywords, countries, and organizations, from 1,465 total documents. Analysis indicates that research has increased recently because of the development of nanotechnologies, particularly through drug delivery and imaging mechanisms. The United States, the People’s Republic of China, and India were the most prevalent publishers, often collaborating with other countries to focus on an integrated global community with similar nanotechnological interests based on shared challenges. Overall, this analysis highlights the continuously evolving field of nano-oncology and how these advancements will reshape global cancer treatment.

## Introduction and background

Life expectancies and cancer rates are directly correlated, exponentially increasing as cancer becomes the second leading cause of mortality worldwide [1]. Due to this positive association, cancer research is increasingly crucial. However, the unpredictability of cancer to specific sites in the body demands various innovative approaches when considering treatment.

Generally, chemotherapy targets all cells within the human body, particularly aimed at inhibiting the proliferation of cancer cells and impairing the ability of healthy cells to reproduce [2]. Radiotherapy offers greater precision by directing beams of radiation towards the tumor. However, this modality poses a risk of damage to surrounding healthy cells [3]. At times, surgery may be considered a safer option by entirely eradicating the malignancy. However, surgery still poses a significant risk of damaging surrounding healthy tissue along with general risks of surgery, particularly associated with organ cancers [4].

While chemotherapy, radiotherapy, and surgery have traditionally been the primary oncological treatments, their toxicity and invasiveness have allowed for nano- and precision technologies to emerge as innovative, minimally invasive interventions [5].

Nanotechnology is the science and engineering of manipulating matter with at least one dimension between 1 and 100 nanometers (nm), where materials exhibit unique physical and chemical behaviors. One nanometer is one-billionth of a meter (10⁻⁹ m); a human hair is about 60,000 nm thick. Specific nanomaterials include nanoparticles and nanotubes [6,7].

Nanoparticles have at least one dimension less than 100 nm. Examples of such particles include gold nanoparticles, quantum dots, and carbon fullerenes. These can have varied shapes, including spherical, tubular, wire-like, or irregular [8]. 

Nanotubes are nanometer-scale tube-like structures, with a hollow, cylindrical shape, like carbon nanotubes. Their walls are just one atom thick, made of carbon atoms in a hexagonal lattice. They are incredibly strong (harder than diamond) yet flexible, with high electrical conductivity. 

Praised for their flexibility and fortitude, nanotubes have a cylindrical shape and a wide array of applications. Particularly due to their low manufacturing costs and minimal‌ side effects on the body, they are more advantageous. These benefits range from drug delivery all the way to biomarker detection [7].

Bibliometric analysis reveals the significance of nanomedical cancer treatment as an increasing phenomenon, focusing on annual publication trends, publication countries and organizations of origin, and keywords frequently associated with the area of study. The research question is what the state of the literature in the field of nano-oncology is. This analysis aims to provide insight into the rapidly evolving oncological field.

## Review

Methods

Bibliometric analysis provides a clear visual of the patterns and trends within a research topic and organizes it to simulate the relations within the literature [9]. The main modes of analysis include the number of published works by country and organization, the number of publications from 2003 to 2025, and keywords relating specifically to nanotechnology in oncology. VOSViewer is the primary tool for analysis, aiding in the visualization of data from Web of Science and providing clear associations and trends between various data points. Web of Science was also utilized for illustrating trends in publication numbers over time. This analysis provides a clear visual of the patterns and trends within a research topic and organizes them to simulate the relationships within the literature [9]. The main modes of analysis include the number of published works by country and organization, the number of publications from 2003 to 2025, and keywords relating specifically to nanotechnology in oncology.

Data Acquisition

**Figure 1:**
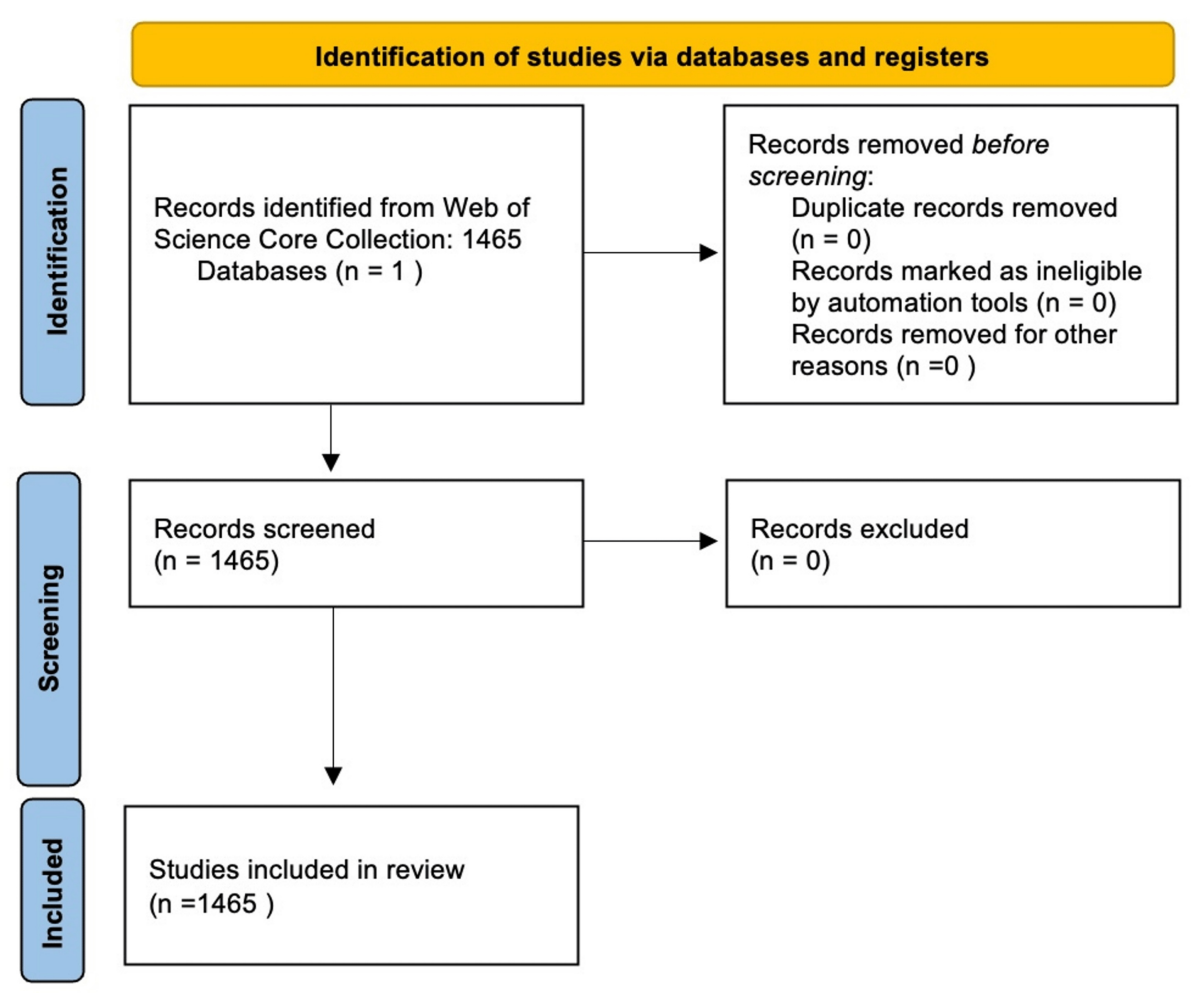
Study selection diagram

The data from this bibliometric analysis was obtained from the Web of Science Core Collection database, utilizing the (((((TS=(oncology)) AND TS=(nanotechnology )) OR (TS=(oncology) AND TS=(nanomedicine)) OR (TS=(oncology) AND TS=(nanoparticle)) OR (TS=(oncology) AND TS=(nanotube))))) commands. The study selection is summarized in Figure 1.

Results

In total, 1465 documents were retrieved for this analysis.

Temporal trends

**Figure 2:**
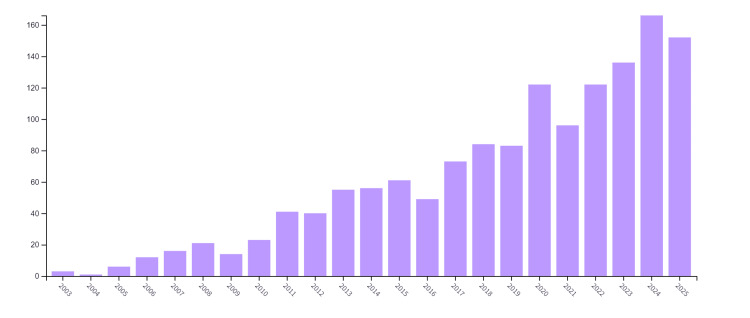
Publication data per publication for oncological nanotechnology research from 2003 to 2025

Figure 2 depicts the steady increase in attention towards the role of nanotechnology in oncology, with the most significant increase from 2023 to 2024 and 2025. Quite remarkably, by June 2025, there have already been 152 publications, suggesting a considerable spike in the number of publications for both this year and onward.

Keyword Analysis

**Figure 3:**
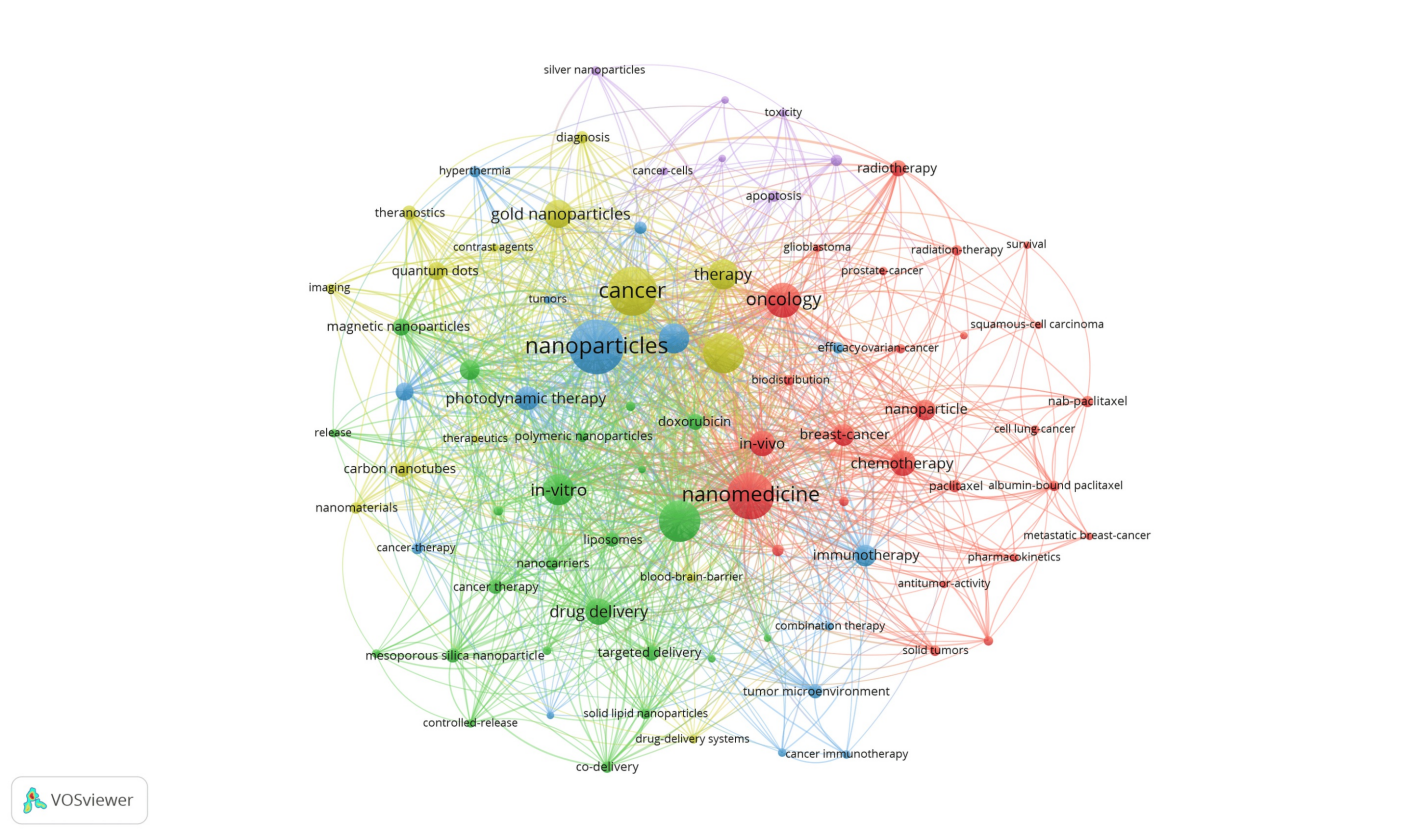
Publication data per keyword for oncological nanotechnology research. Keywords mentioned frequently are larger circles, webbing connecting terms often linked together

**Figure 4:**
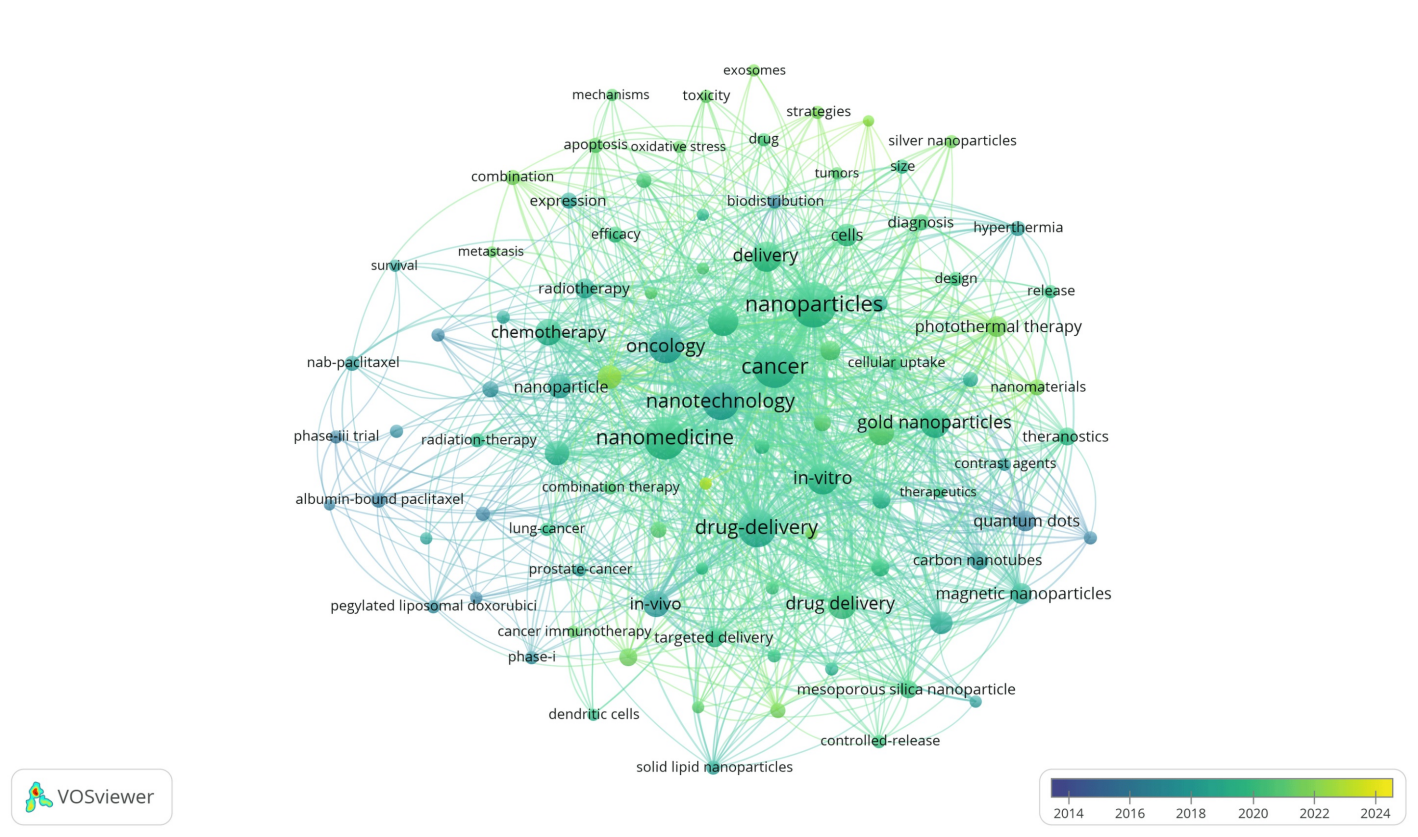
Publication data per keyword for oncological nanotechnology research from the last full ten years. Keywords more recently (such as 2023 or 2024) have a yellow hue, while those more distant (such as 2014) have a darker blue hue

Figures 3, 4 both illustrate essential terms within nano-oncological research, where larger circles represent more frequently included words and lines associating keywords together. Figure 3 displays central keywords, such as nanotechnology/nanomedicine with a combined total of 540 occurrences, cancer/oncology with a combined occurrence number of 515, and nanoparticles with 366 occurrences. Keywords more specific to nano-oncological research provide greater insight into the types of nanotechnology and the role they serve, including terms such as drug delivery (and alternatively, drug-delivery) with a total of 386 occurrences, gold nanoparticles with 151 occurrences, and iron-oxide nanoparticles with 93 occurrences. Figure 4 more evidently presents the evolution of terminology in oncological research, where words shaded closer to yellow represent more recent utilization and reflect altering interventions in the field. As innovation continues, nanotechnologies are expected to advance, leading to new discoveries and increased reference to emerging key terms, while traditional treatments, such as chemotherapy, may appear more frequently in earlier years.

Geographical Distribution

**Figure 5:**
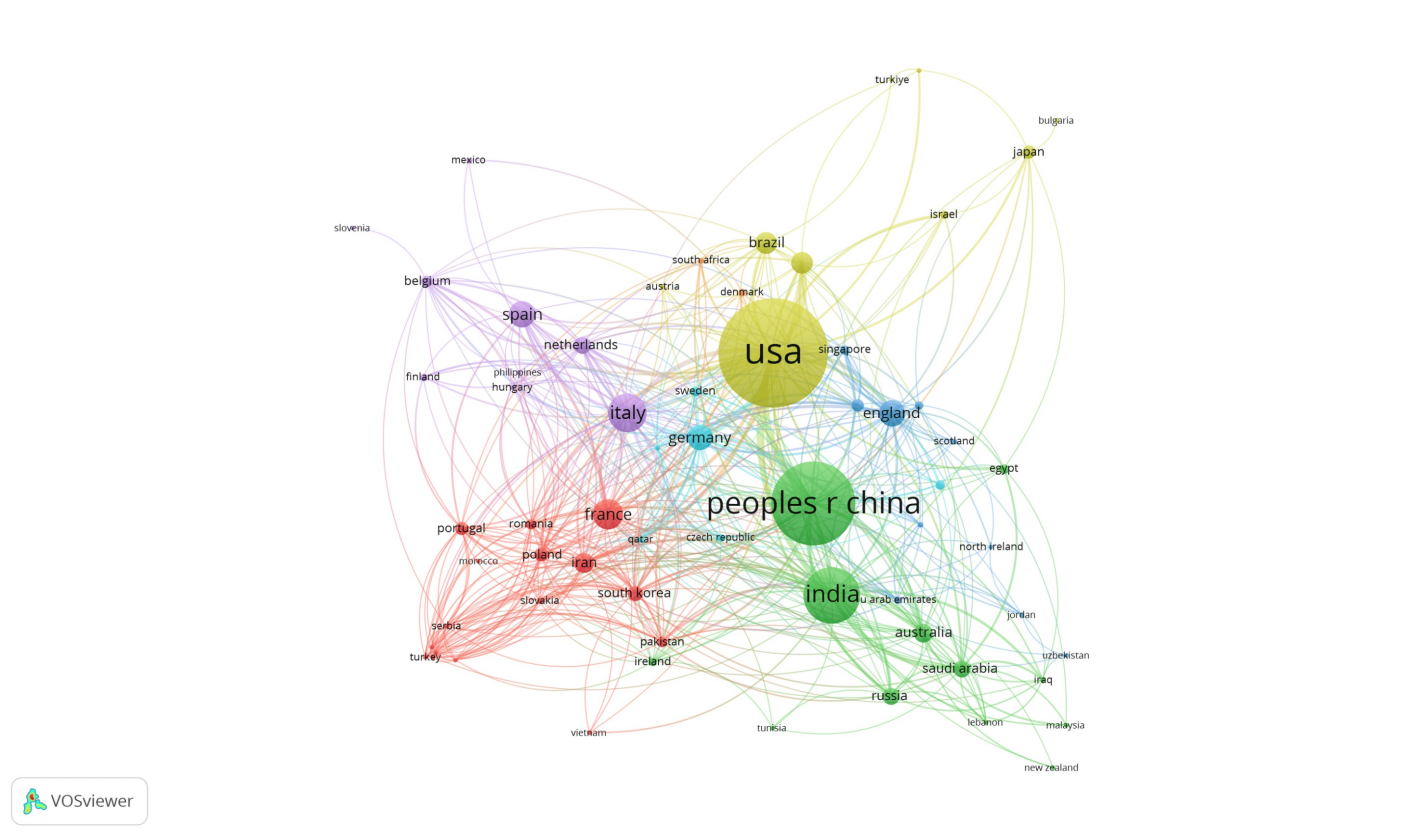
Publication data per country for oncological nanotechnology research

**Figure 6:**
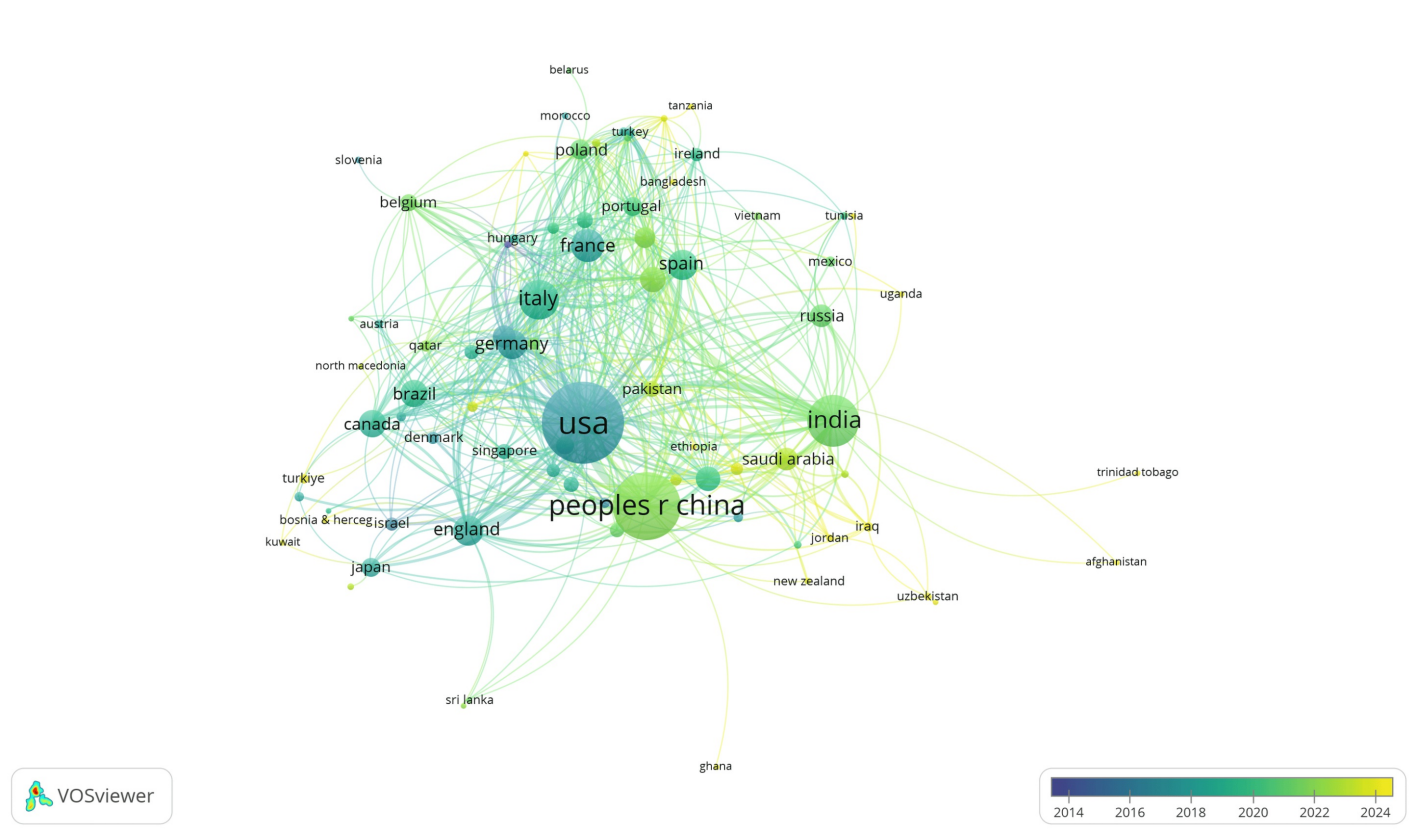
Publication data per country for oncological nanotechnology research from the past ten full years of data

**Figure 7:**
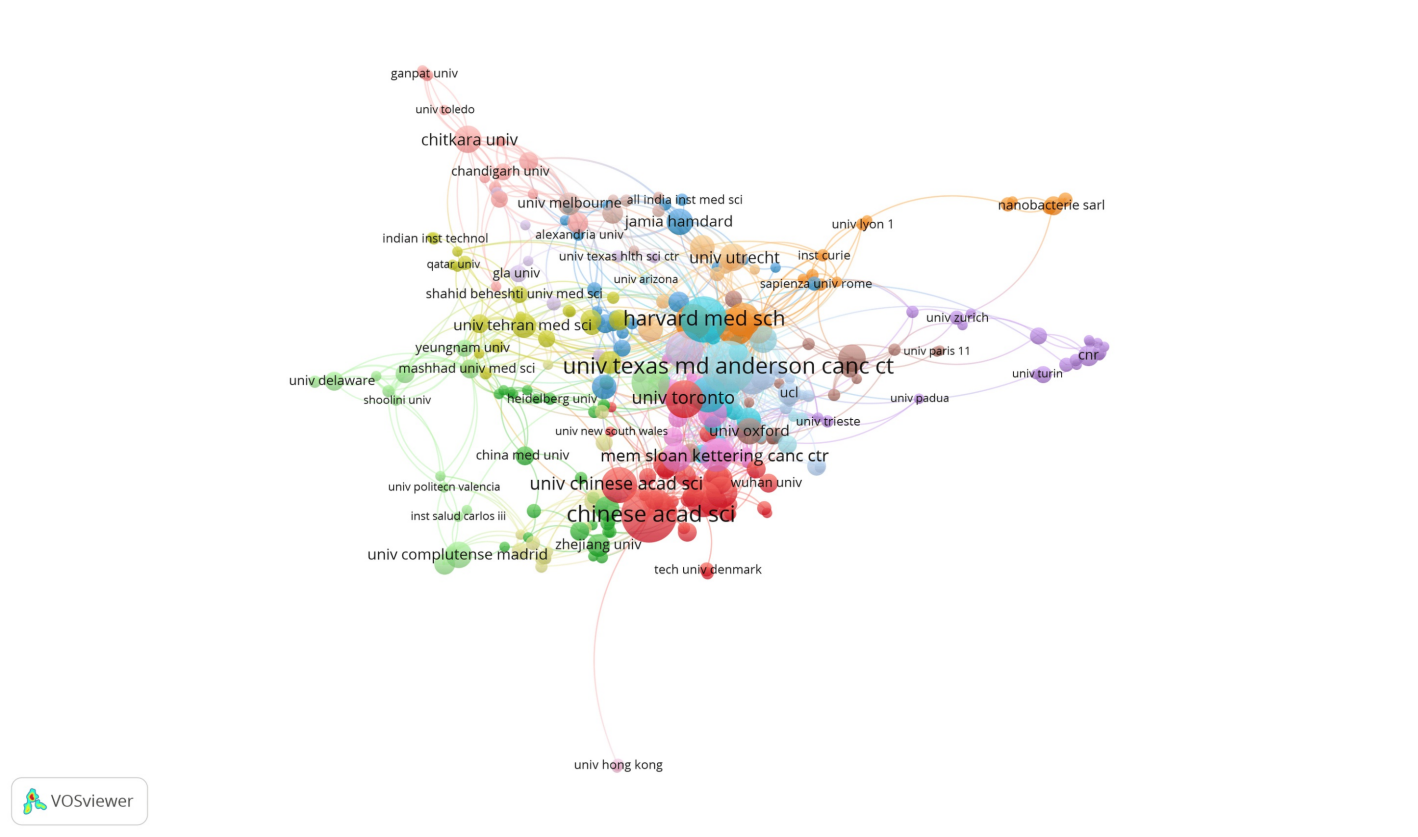
Publication data per organization for oncological nanotechnology research

Figures 5-7 relate countries, publication dates by country, and organizations through illustrated lines, respectively. Figure 5 depicts that most research in oncological nanotechnology is connected to three major countries known for their scientific research, namely the United States (449 documents), the People’s Republic of China (PRC) (312 documents), and India (182 documents). These geographic regions not only have robust scientific programs, but also harbor a significant portion of the world’s population. Based upon this evidence, it is unsurprising that most documents are from highly populated countries. Figure 6 illustrates how more recent publications are related to developing countries who primarily collaborate with the United States, the PRC, and India. Figure 7 focuses on specific organizations, most from the aforementioned major countries, such as the Chinese Academy of Sciences (31 documents), University Texas MD Anderson Cancer Center (29 documents), and Harvard Medical School (24 documents). Most are from reputable universities, while others are from cancer research institutes, including the National Cancer Institute (NCI) (18 documents) and the Memorial Sloan Kettering Institute for Cancer Research (15 documents).

Discussion

International Collaborations

This study examined 1,465 publications from 2003 to 2025. The countries with the largest role in nano-oncological research were as follows: the United States, the PRC, and India. These countries account for the majority of publications in the nano-oncological field, as shown in Figure 5. However, each of these countries has varying spheres of influence and collaborations. As one of the major research-producing countries in North America, the United States has significantly greater influence over all continents, reinforcing its consideration as a world power. The United States collaborates with countries beyond its geographic borders. This is further facilitated by its diplomatic relations [10]. The PRC, on the other hand, has predominantly worked with European countries and select Middle Eastern or African countries. Within Asia, the PRC has comparatively limited research collaboration with neighboring countries [11]. Comparatively, India stays within its geographical sphere, focusing mostly on Asia, the Middle East and Africa, and even East Europe. This is particularly due to its historical, cultural, and political ties, allowing India to prioritize more immediate neighbors [12].

However, most research reflects the application of nanotechnology in developed countries, particularly the United States and Europe. Of those that are published, developing countries have predominantly written reviews and co-authored with one of the three leading nations: the United States, the PRC, or India.

As illustrated in Figure 6, the oldest publications are from high-income countries (closer to 2014 on the timeline), whereas newer publications are from low-income countries (closer to 2024 on the timeline), and those in the middle are typically middle-income countries, which may correlate with the development of infrastructure and awareness of nanotechnology [13].

In some cases, developing countries have distinct resources that enable specific topics, depending on local conditions. For example, a study utilized the plant protease papain from the papaya to produce protein nanoparticles to deliver drugs [14]. The papaya is typically native to tropical areas, so research would be central to these regions, such as Brazil [15].

Health Disparities

However, the nanotechnological research in middle- and low-income countries can often revolve around disparities in these countries. While there are more instances of prostate cancer in the West, patients in the Middle East tend to get diagnosed later in life. Subsequently, they may advance to a later stage in the disease progression with a greater increase in mortality-to-incidence rate [16,17]. Of note, prostate cancer is relatively well recognized in the United States, but there is less knowledge and public awareness in the North Africa/Middle East region, corresponding to increased mortality [16].

Clinical Applications

**Figure 8:**
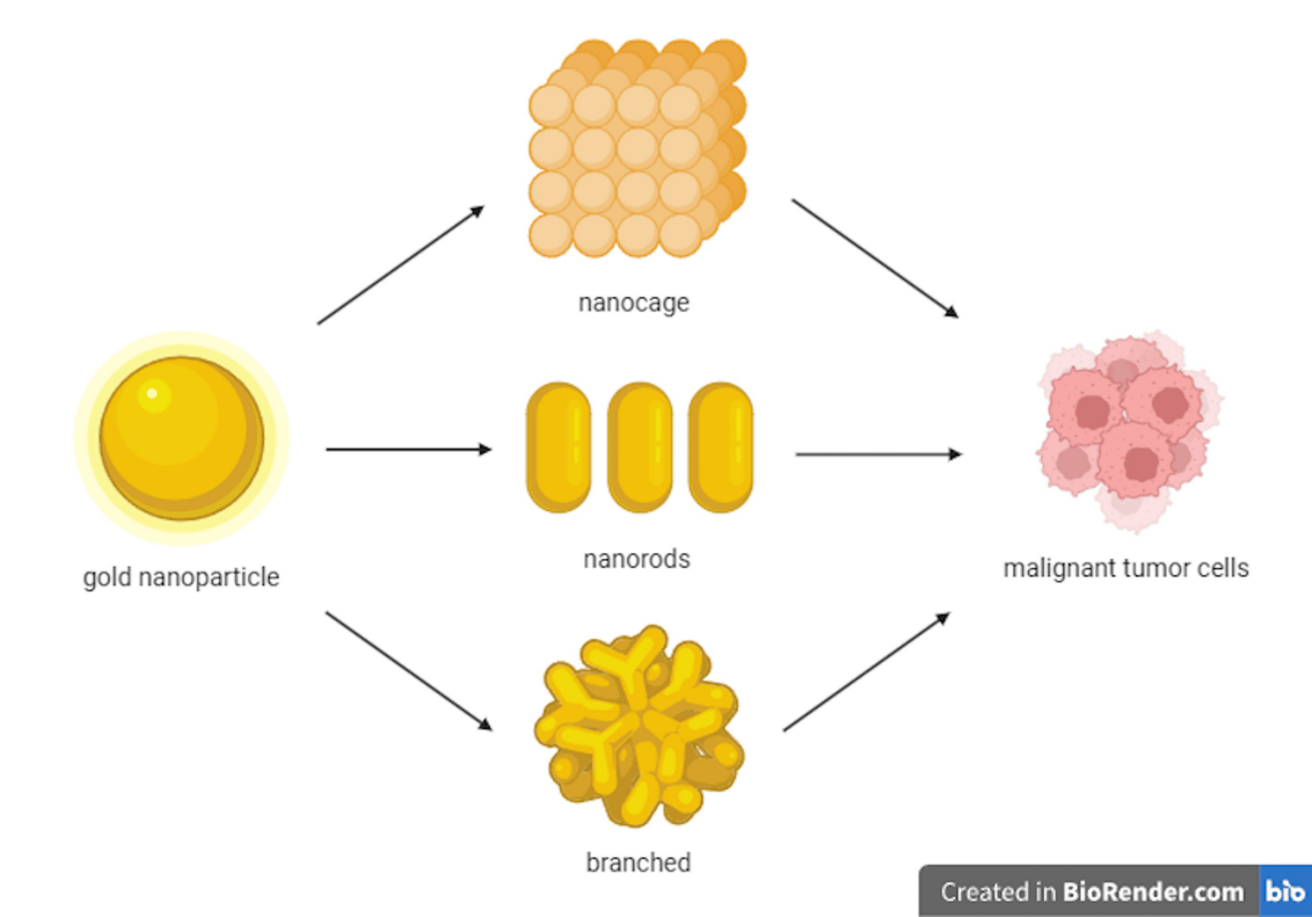
Differently shaped and structured gold nanoparticles correlate to different functions with regard to malignant tumor cells. Created by Tori Baer in BioRender.com

In this case, gold nanoparticles were utilized to provide a less invasive and safer form of cancer treatment: photothermal therapy. When light encounters the free electrons on the nanoparticle’s surface, the light scatters, allowing for specific wavelengths to be absorbed [18,19]. They in turn emit electron signals, which can be then used to identify certain molecules, such as DNA or proteins [18]. Depending on their shape and structure (such as nanorods, nanocubes, nanospheres, and nanostars; depicted in Figure 8), the types of wavelengths they absorb inversely changes. This allows them to emit heat onto targeted malignant tumor cells, which can then lead to the destruction of the cancer [19]. Especially with larger surface areas of prostate cancer, this therapy can retain as many healthy cells as possible and provide a safer treatment without the risks of surgeries for radical removal of tissue. This will also allow for a reduction of migration to surrounding tissue and cause side effects to healthy cells [20].

**Figure 9:**
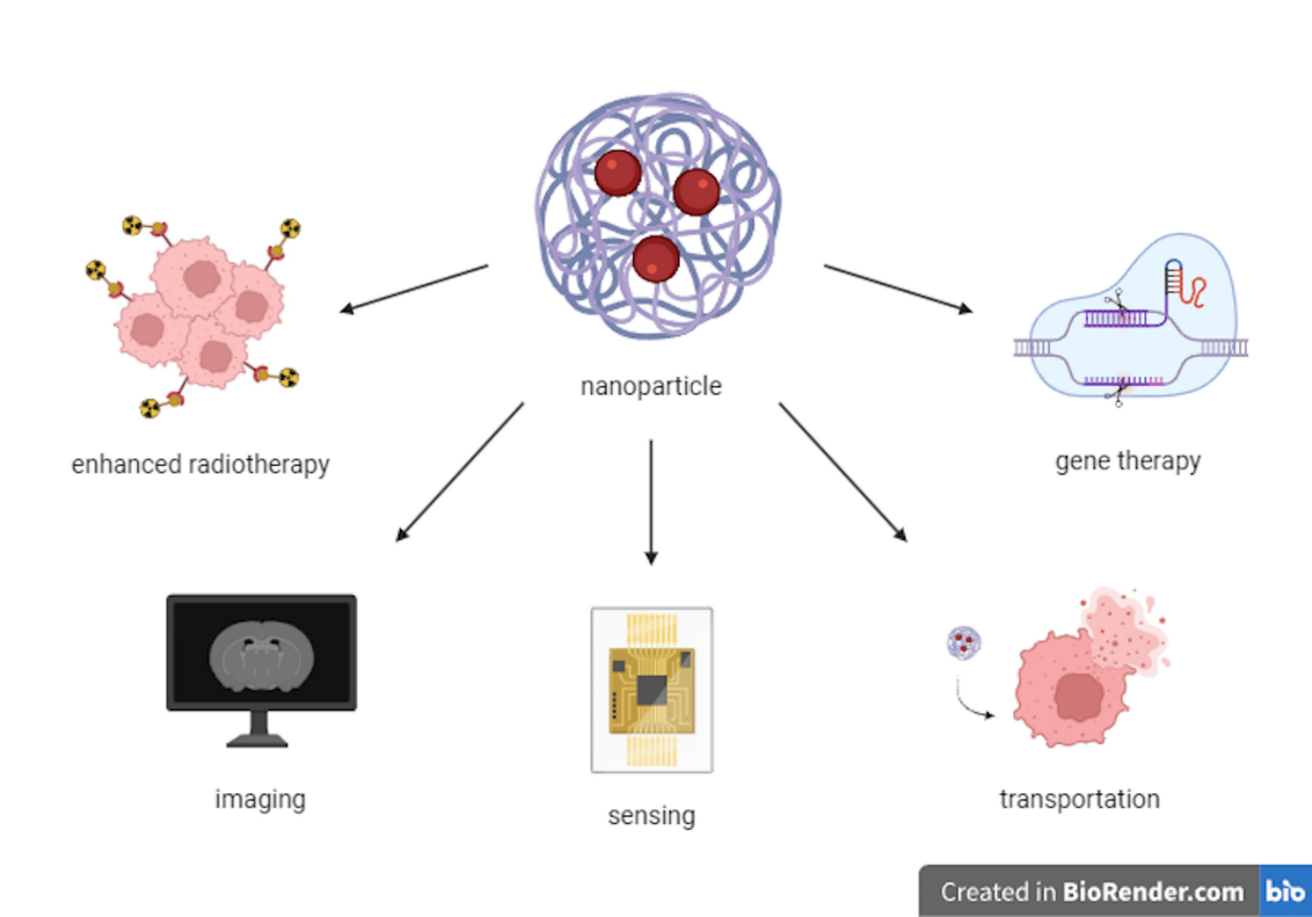
Nanoparticles have many applications, depending on the type of nanoparticle and the specific conditions Created by Tori Baer in BioRender.com

In general, within nano-oncology, nanotubes and nanoparticles continue to garner more attention due to their unique properties, consideration of the location of the patient’s tumor, medication release times (including controlled release), and ability to transport medication directly to the tumor site. Drug delivery specifically has 386 occurrences, as depicted in Figure 9, which suggests the primary function of nanotechnology (such as through nanotubes) is by transporting drugs. Lipid nanoparticles (26 occurrences) are one of the most favored forms of nano-transportation and includes solid lipid nanoparticles with 37 occurrences, nanostructure lipid carriers, liposomes, and noisomes. They are notably inexpensive, easily producible, and permeable through various tough membranes [21]. Certain nanoparticles, such as iron-oxide nanoparticles and quantum dots, can also be used for imaging due to their specificity and highly permeable fluorescence, respectively [22,23].

The versatility of nanoparticles allows for region-specific innovations driven by local conditions, which is where international partnership becomes particularly valuable. Countries often collaborate due to shared concerns and perspectives, promoting their integration. One particular study highlights the use of algae to synthesize metallic nanoparticles, a green alternative, with anti-cancerous properties that can be used for drug delivery and imaging [24]. The collaborators are from Suzhou, PRC; La Jolla, California, United States; Islamabad, Pakistan; and Rajagiriya, Sri Lanka, which are (except Islamabad) near major bodies of water and sources of algae. Of importance, these areas face serious environmental concerns. The Suzhou region is infiltrated with heavy metal pollution in the soil, which can negatively affect food quality [25]. California faces severe wildfire and air pollution issues. Due to climate change and subsequent air pollution, Pakistan experiences unusual weather patterns, such as flooding and droughts [26]. Sri Lanka suffers from poor air pollution as a result of factory emissions and agricultural burns, which carry toxic chemicals and carcinogens [27]. Overall, these regions are willing to explore environmentally friendly nanotechnologies, fostering collaborations with varying innovations in other areas.

As illustrated in Figure 7, most institutions engaged in nano-oncological research include large universities, particularly located in the United States and the PRC. These institutions often contain numerous laboratories and are adequately funded by both the university and government. These universities encourage research endeavors by offering essential equipment to foster an accessible environment where students can collaborate on shared research interests. Cancer institutions such as the Memorial Sloan Kettering Cancer Center, which are dedicated to oncological research, have also demonstrated significant influence in advancing nano-oncological research. Global patterns in nano-oncology highlight major advancements in biomedical research, shaped by geographic distribution, international collaborations, and the significant influence and contributions of key institutions.

Limitations

This study only focused on data readily available in the Web of Science Core Collection, which may have excluded other relevant publications. A comprehensive search utilizing other databases, such as Scopus or PubMed, would provide a more detailed and accurate depiction of nanoparticles and nanotubes in oncology. Additionally, geographical bias was also observed with most of the data retrieved from the United States and the PRC. As a result, cancers considered less critical in more developed countries may be significantly more fatal in less developed regions. Research focused on these cancers and their accompanying nanoparticles hold heightened significance in countries experiencing greater health disparities. Language bias was also present as only publications written in English were included, potentially creating gaps in data exportation, especially concerning research discussing advancements in nano-oncological care from non-English speaking countries. Future bibliometric studies can overcome these limitations to produce a more comprehensive view of the literature in the field of nano-oncology.

## Conclusions

This bibliometric analysis illustrates the expansion and diversification of nanoparticle and nanotube research within nano-oncology, where nanoparticles continue to be modified for varying therapeutic and diagnostic applications. While the United States, the PRC, and India are the leading contributors to literature, collaborations with developing countries drive regional concerns and priorities to the forefront for a united approach to cancer treatment, revolutionizing the field. As concerns surrounding cancer continue to persist, especially with regard to rising life expectancy, the continued advancement of nanotechnologies for cancer treatment grows urgent, reiterating the critical need for international collaboration to improve the effectiveness of cancer care.
